# Pulmonary Fibrosis and Hypereosinophilia in TLR9^-/-^ Mice Infected by *Cryptococcus gattii*

**DOI:** 10.3390/pathogens11090987

**Published:** 2022-08-29

**Authors:** Elias Barbosa da Silva-Junior, Israel Diniz-Lima, Amanda Couto Silva, Joyce Cristina Guimarães-de-Oliveira, Alexandre Morrot, Leonardo Freire-de-Lima, Leonardo Marques da Fonseca, Lycia de Brito-Gitirana, Debora Decote-Ricardo, Herbert Leonel de Matos Guedes, Celio Geraldo Freire-de-Lima

**Affiliations:** 1 Instituto de Biofísica Carlos Chagas Filho, Universidade Federal do Rio de Janeiro, Rio de Janeiro 21941-900, Brazil; 2 Instituto de Veterinária, Universidade Federal Rural do Rio de Janeiro, Seropédica 23890-000, Brazil; 3 Instituto Oswaldo Cruz, FIOCRUZ, Rio de Janeiro 21045-900, Brazil; 4 Faculdade de Medicina, Universidade Federal do Rio de Janeiro, Rio de Janeiro 21941-900, Brazil; 5 Instituto de Ciências Biomédicas, Universidade Federal do Rio de Janeiro, Rio de Janeiro 21941-900, Brazil; 6 Instituto de Microbiologia Paulo de Góes, Universidade Federal do Rio de Janeiro, Rio de Janeiro 21941-900, Brazil

**Keywords:** *Cryptococcus gattii*, toll-like receptor 9, eosinophilia, infection, histopathological analyses

## Abstract

*Cryptococcus gattii* is a worldwide-distributed basidiomycetous yeast that can infect immunocompetent hosts. However, little is known about the mechanisms involved in the disease. The innate immune response is essential to the control of infections by microorganisms. *Toll*-like receptor 9 (TLR9) is an innate immune receptor, classically described as a non-methylated DNA recognizer and associated with bacteria, protozoa and opportunistic mycosis infection models. Previously, our group showed that TLR9^-/-^ mice were more susceptible to *C. gattii* after 21 days of infection. However, some questions about the innate immunity involving TLR9 response against *C. gattii* remain unknown. In order to investigate the systemic cryptococcal infection, we evaluated C57BL/6 mice and C57BL/6 TLR9^-/-^ after intratracheal infection with 10^4^ *C. gattii* yeasts for 21 days. Our data evidenced that TLR9^-/-^ was more susceptible to *C. gattii.* TLR9^-/-^ mice had hypereosinophilia in pulmonary mixed cellular infiltrate, severe bronchiolitis and vasculitis and type 2 alveolar cell hyperplasia. In addition, TLR9^-/-^ mice developed severe pulmonary fibrosis and areas with strongly birefringent fibers. Together, our results corroborate the hypothesis that TLR9 is important to support the Th1/Th17 response against *C. gattii* infection in the murine experimental model.

## 1. Introduction

*Cryptococcus gattii* is a pathogenic encapsulated basidiomycete widely distributed in the environment and capable of infecting immunocompetent individuals, mainly affecting the lungs and central nervous system (CNS) [1,2,3]. *C. gattii* is saprophytic and can adapt to the most diverse environments and was the main etiological agent in the North Pacific outbreak that occurred in 1990s [4,5,6]. *C. gattii* and *C. neoformans* yeasts are the only pathogenic encapsulated fungi. Glucuronoxylomannan (GXM) and glucuronoxylomannogalactan (GXMGal) polysaccharides are the main components of the capsule that covers the yeast and represent two of the most important virulence factors, although other virulence factors such as melanin production, abnormally sized yeast (titan cells), vesicle release and growth at 37 °C are also important and well-described in the literature [7,8,9,10,11,12,13]. The transmission can occur through the inhalation of basidiospores or yeast cells that are easily deposited in the alveoli, initially damaging the respiratory system and being able to spread to other organs, such as the brain [1,2,3].

Unlike *C. neoformans*, *C. gattii* can infect healthy individuals, and the host Th1 and Th17 effector responses are deficient and do not contribute to the control of fungal spread [14,15]. Some recent studies have shown the importance of the Th1 and Th17 effector response against *Cryptococcus* spp. Early and late IL-10 blockade enhanced the Th1 and Th17 effector response in C57BL/6 mice infected with *C. neoformans*, leading to fungal clearance [16]. A vaccination model using antigen-primed dendritic cells was able to induce a Th1 and Th17 effector response against *C. gattii*, decreasing the fungal spread and improving the survival rate of mice after pulmonary infection [17]. Dendritic cells seem to be the main route of antigen presentation for T lymphocyte stimulation against *Cryptococcus* spp. [18]. Jamil and col. (2020) showed, in vitro, that *C. gattii* yeasts caused immunoparalysis in human dendritic cells (DCs) differentiated from monocytes [19]. By preventing phagosome maturation, yeasts induced low expression of surface and internal markers in DCs such as CD80, CD86, HLA-2, IFN-γ and IL-8.

Recently, our group demonstrated that BALB/c X-linked immunodeficient mice (XID) were more susceptible to *C. gattii*, indicating an important role of B cells during experimental infection, producing lower amounts of anti-GXM antibodies, IL-17 and IFN-γ in comparison to WT infected mice [20]. In addition, we also showed that cryptococcal capsular polysaccharides can induce macrophage apoptosis [21] via FasL and downregulate NET production in vitro [22]. However, type 2 alveolar epithelial cells and eosinophils may play an important role in cryptococcal pneumonia [23,24,25,26]. Type 2 alveolar epithelial cells are a major source of IL-33, amplifying the Th2 immune response and supporting prolonged and disseminated cryptococcal pneumonia in the course of experimental infection using *C. neoformans* [23,27,28]. Many works have demonstrated that cryptococcal pneumonia leads to an intense and prolonged pulmonary Th2 inflammatory response. The “allergy” response is like other chronicle inflammatory diseases, such as asthma and allergic bronchopulmonary mycosis (ABPM) [29,30,31,32].

TLR9 is an innate immune receptor expressed and coupled to endosomal vesicles, mainly on plasmocytoid dendritic cells and B cells. Classically, TLR9 can recognize CpG motifs in non-methylated single-stranded DNA (ssDNA), which is commonly found in bacteria and protozoa [33,34]. The presence of repeated sequences of non-methylated CpG dinucleotides allows the interaction of TLR9 with ssDNA, leading to translocation from the endoplasmic reticulum towards lysosomes and the Golgi complex [35]. The intracellular pathway activates dendritic cells, macrophages and B cells through recruitment and interaction with MyD88, leading to cytokine, chemokine and immunoglobulin production. Some studies have shown that TLR9 plays an important role in the control of fungal infections caused by *Aspergillus fumigatus* [36], *Candida albicans* [37] and *C. neoformans* [38,39]. Previously, our group showed that in vitro *C. neoformans*-infected DCs were able to produce IL-12 and IL-6, inducing the differentiation of naïve T cells into Th1 and Th17 effector T cells [40]. More recently, we demonstrated that TLR9-deficient mice were more susceptible to *C. gattii* hypervirulent strain R265 in an intratracheal infection model [41]. However, the mechanisms involved in the TLR9 response against *C. gattii* are still poorly understood.

In the current study, we compared C57BL/6 and C57BL/6 TLR9^-/-^ mice infected with *C. gattii* hypervirulent strain R265 21 days after inoculum. Some studies have demonstrated a Th2/allergic immune response in detriment of a protective Th1/Th17 immune response in experimental cryptococcosis. Therefore, we evaluated aspects related to a Th2/allergic immune response in the presence or absence of TLR9 during experimental infection with *C. gattii*. Our results suggest that TLR9^-/-^ infected mice had intense pulmonary fibrosis, hypereosinophilia and type 2 epithelial alveolar cell hyperplasia.

## 2. Results

### 2.1. Intratracheal Infection by C. gattii Causes a Severe Lung Injury with Epithelial Destruction

**Figure 1:**
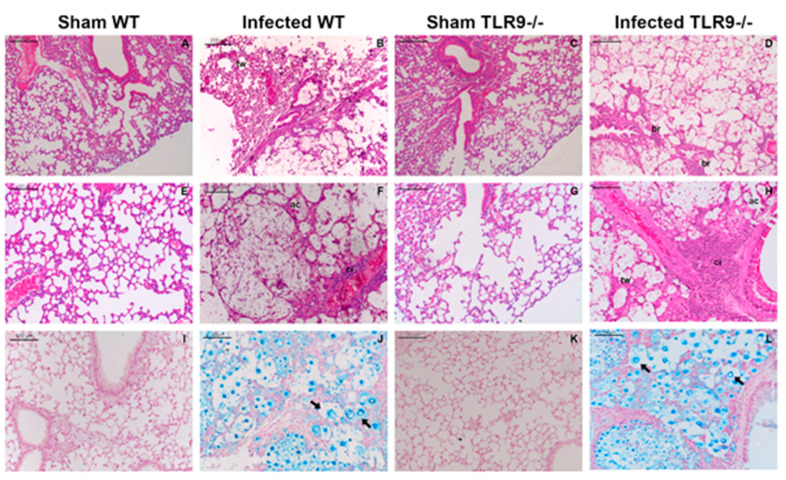
Pulmonary impairment during experimental *C. gattii* infection. WT and TLR9^-/-^ mice intratracheally received 10^4^ *C. gattii* yeasts suspended in 30 µL of PBS. A total of 21 days after inoculum, both groups were euthanized, and the organs were collected and prepared for histology as described in Methods. Lung histological sections from Sham WT mice (**A**) (*n* = 3), *C. gattii*-infected WT mice (**B**) (*n* = 4), Sham TLR9^-/-^ mice (**C**) (*n* = 3) and *C. gattii*-infected TLR9^-/-^ mice (**D**) (*n* = 3) stained with hematoxylin and eosin. Infected WT mice had a higher frequency of alveolar wall thickening (**B**). Severe bronchiolitis was observed in the lungs of infected TLR9^-/-^ mice (**D**). Lung histological sections from Sham WT mice (**E**), *C. gattii*-infected WT mice (**F**), Sham TLR9^-/-^ mice (**G**) and *C. gattii*-infected TLR9^-/-^ mice (**H**) stained with hematoxylin and eosin. Histological sections at higher magnification show alveolar congestion and cellular infiltrate in both infected WT (**F**) and TLR9^-/-^ (**H**) mice, although the TLR9^-/-^ infected mice had a robust cellular infiltrate. Lung histological sections from Sham WT mice (**I**), *C. gattii*-infected WT mice (**J**), Sham TLR9^-/-^ mice (**K**) and *C. gattii*-infected TLR9^-/-^ mice (**L**) stained with Alcian Blue. Black arrows indicate *C. gattii* titan cells. Magnification ×10 (**A**–**D**); magnification ×20 (**E**–**L**).

Both WT and TLR9^-/-^ mice had their lungs severely injured after 21 days of *C. gattii* infection. Atelectasis, alveolar congestion, hemorrhage, increased macrophage aggregation, cellular infiltrate and thickening of alveolar walls were observed in the lungs of both infected groups 21 days after *C. gattii* inoculum (Figure 1B,H). However, TLR9^-/-^ mice showed generalized alveolar destruction, intense cellular infiltrate and epithelial disintegration with loss of bronchial epithelial cells (Figure 1D,H). Curiously, in a previous study, our group showed that there was no difference between TLR9^-/-^ and WT mouse lung CFU 21 days post *C. gattii* infection, as well as a higher frequency of titan cells in the lungs, evidencing an increased susceptibility to *C. gattii* [41]. Recently described, titan cells are frequently associated with the Th2 immune response and the increased susceptibility of mice in cryptococcal experimental models, as well as with in vitro infection assays that have shown the immunomodulatory function of these cells [42,43,44,45]. As described in our previous research, *C. gattii* titan cells were observed in the lungs of both WT and TLR9^-/-^ mice (Figure 1J,L).

### 2.2. TLR9^-/-^ Infected Mice Have Hypereosinophilia in Mixed Inflammatory Infiltrate

**Figure 2:**
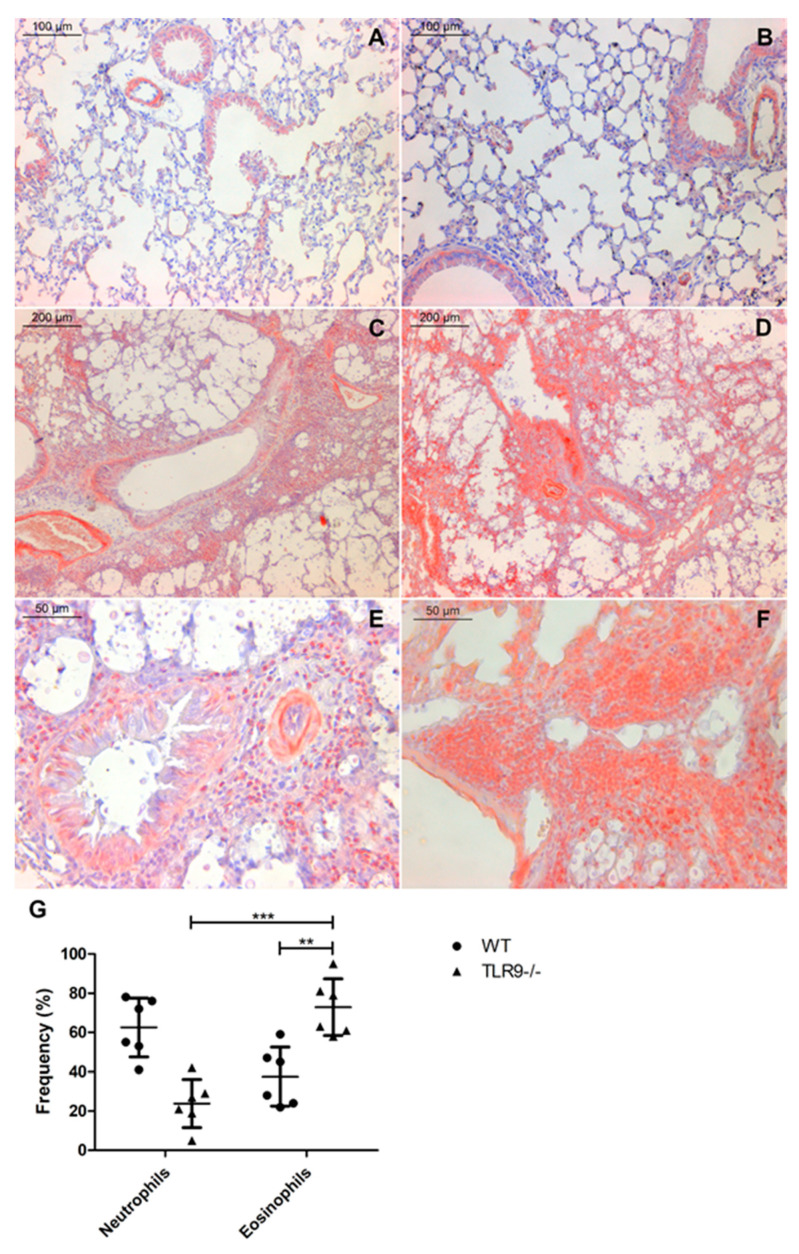
Hypereosinophilia in TLR9^-/-^ during *C. gattii* infection. WT and TLR9^-/-^ mice intratracheally received 10^4^ *C. gattii* yeasts suspended in 30 µL of PBS. A total of 21 days after inoculum, both groups were euthanized, and the organs were collected and prepared for histology as described in Methods. Representative lung histological sections from Sham WT mice (**A**) (*n* = 3), Sham TLR9^-/-^ mice (**B**) (*n* = 3), *C. gattii*-infected WT mice (**C**,**E**) (*n* = 6) and *C. gattii*-infected TLR9^-/-^ mice (**D**,**F**) (*n* = 6) stained with Congo red. TLR9^-/-^ infected mice had a higher frequency of eosinophils (**D**,**F**) than WT infected mice (**C**,**E**). Magnification ×10 (**C**,**D**); magnification ×20 (**A**,**B**); magnification ×40 (**E**,**F**). Frequency of neutrophils and eosinophils in pulmonary mixed infiltrate (**G**). Each neutrophil and eosinophil were counted to estimate the frequency as described in Methods. Student’s *t*-test: ** *p* ≤ 0.001; *** *p* ≤ 0.0001.

Polymorphonucleated (PMN) cell infiltrates are a common feature of lung infections caused by microorganisms, such as *C. neoformans* and *C. gattii* [24,25,46,47,48]. Neutrophils and eosinophils are the most important leukocytes in asthma and allergic bronchopulmonary mycosis (ABPMs) [32]. Although they are few in number in circulating blood and lungs of healthy individuals [49], eosinophils are more frequent in allergic asthma and allergic bronchopulmonary aspergillosis (ABPA), as well as the Th2 cytokine repertory, comprised principally by IL-5 and IL-13 [50]. Additionally, the Th1/Th17 immune response was reduced in a murine model of *C. gattii* experimental infection, while expression of Th2 profile cytokines was increased [15]. PMN cells, as expected, were present in large numbers in the lungs of both infected mice groups. However, eosinophils were more frequent in the lungs of infected TLR9^-/-^ mice. A total of 21 days after *C. gattii* infection, TLR9-deficient mice had a large number of PMN cells on the mixed inflammatory infiltrate, as well as close to the vessel wall (lumen) and bronchioles—indicating bronchiolitis (Figure 1H). Furthermore, in order to determine the frequency at which neutrophils and eosinophils appear in the cellular infiltrate, we stained the histological sections with Congo red (Figure 2).

**Figure 3:**
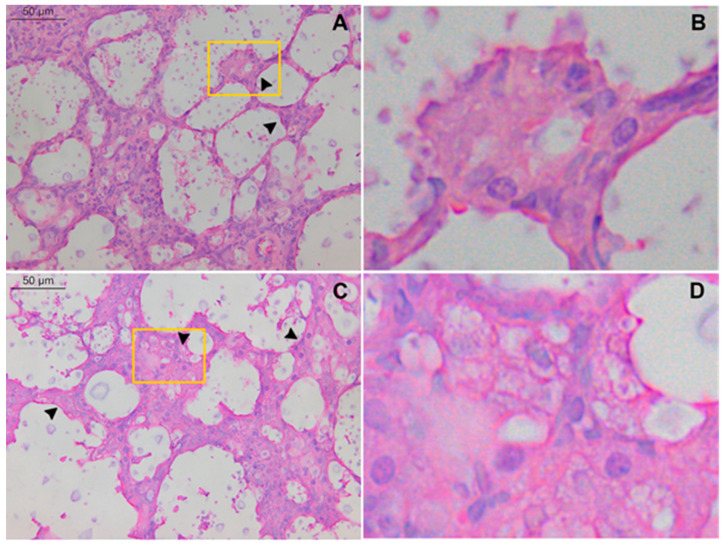
Type 2 alveolar epithelial cell hyperplasia during *C. gattii* infection. WT and TLR9^-/-^ mice intratracheally received 10^4^ *C. gattii* yeasts suspended in 30 µL of PBS. A total of 21 days after inoculum, both groups were euthanized, and the organs were collected and prepared for histology as described in Methods. Representative lung histological sections from *C. gattii*-infected WT mice (**A**,**B**) (*n* = 4) and *C. gattii*-infected TLR9^-/-^ mice (**C**,**D**) (*n* = 3) stained with hematoxylin and eosin. Large number of epithelial alveolar cells clustered in thickened alveolar walls. In addition, PMN cells, alveolar macrophages and *C. gattii* titan cells were observed. Histological sections at higher magnification showed type 2 alveolar epithelial cells from both infected WT (**B**) and TLR9^-/-^ (**D**) mice. Black arrowheads indicate type 2 alveolar epithelial cell hyperplasia. Magnification ×40 (**A**,**C**).

Eosinophils were more frequent in mixed inflammatory infiltrate from TLR9^-/-^ infected mice than WT infected mice 21 days after infection. Histological sections showed that TLR9^-/-^ mice had pulmonary hypereosinophilia 21 days after inoculum (Figure 2D,F). In addition, TLR9^-/-^ infected mice had a higher frequency of eosinophils than neutrophils in mixed cellular infiltrate (Figure 2G). Some studies have already shown that IL-33 plays an important role in the pulmonary Th2 profile, activating type 2 innate lymphoid cells (ILC2) and increasing eosinophilia [51,52]. Interestingly, Heyen and col. (2016) showed that type 2 alveolar epithelial cells are the main source of IL-33 in the lungs during *C. neoformans* infection. However, the role of IL-33 during *C. gattii* infection is still poorly understood. Our results showed a high frequency of hyperplasic type 2 alveolar epithelial cells in both WT and TLR9^-/-^ mice 21 days after *C. gattii* infection (Figure 3C,D). This result may indicate a crucial role of type 2 alveolar epithelial cells during *C. gattii* infection.

### 2.3. Large Fibrosis in the Lungs of TLR9^-/-^ Infected Mice

**Figure 4:**
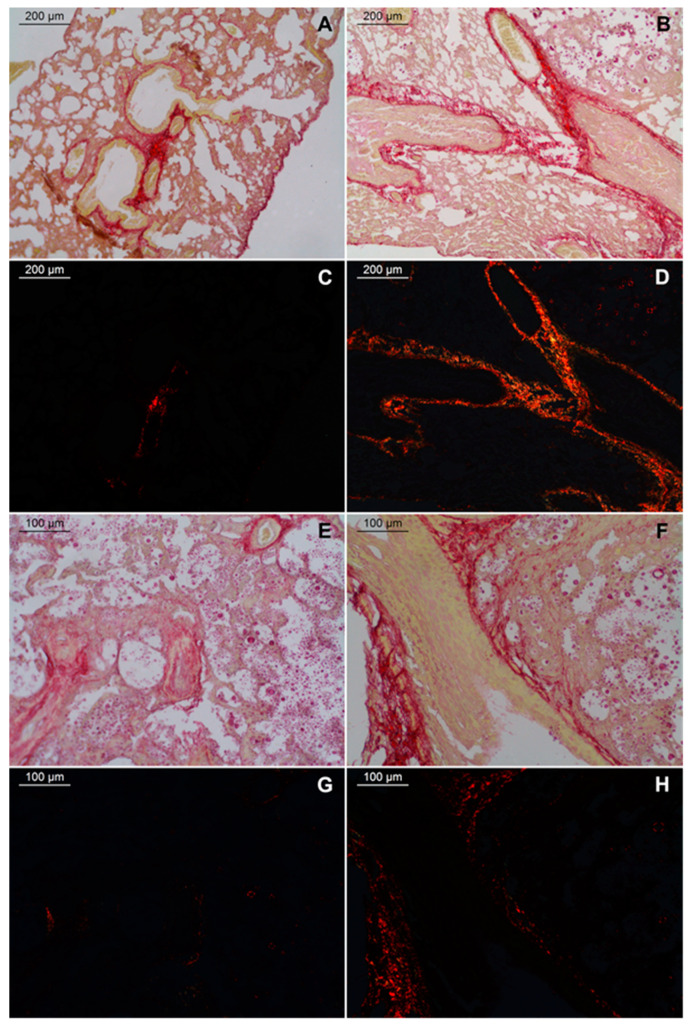
TLR9^-/-^ mice had large fibrosis during *C. gattii* infection. WT and TLR9^-/-^ mice intratracheally received 10^4^ *C. gattii* yeasts suspended in 30 µL of PBS. A total of 21 days after inoculum, both groups were euthanized, and the organs were collected and prepared for histology as described in Methods. There was a predominance of fibrous elements stained in red. Intense pulmonary fibrosis was seen in the lungs of TLR9^-/-^ infected mice, especially around the arterioles and bronchioles. Representative lung histological sections from *C. gattii*-infected WT mice (left column) (*n* = 3) and *C. gattii*-infected TLR9^-/-^ mice (right column) (*n* = 3) stained with picrosirius red and viewed by bright-field microscopy (**A**,**B**,**E**,**F**) or with linear polarization (**C**,**D**,**G**,**H**). Magnification ×10 (**A**,**D**); magnification ×20 (**E**,**H**).

Fibrosis is another aspect resulting from chronic lung inflammation. To investigate fibrosis in the lungs of C57BL/6 mice infected by *C. gattii*, we stained the histological sections with picrosirius red. We observed areas with a predominance of fibrous elements in the lungs of both WT and TLR9^-/-^ infected groups 21 days after inoculum (Figure 4). However, thick red, strongly birefringent fibers were clearly observable in the lungs of TLR9-deficient mice (Figure 4D,H). Areas with large fibrosis were concentrated around vessels, arterioles and bronchioles of both WT and TLR9^-/-^ infected mice (Figure 4A,B,E,F). In addition, TLR9^-/-^ infected mice had small sites where weakly birefringent orange-green fibers were concentrated (Figure 4D). Interestingly, type 2 alveolar epithelial cell hyperplasia was associated with idiopathic pulmonary fibrosis [53].

## 3. Discussion

In this work, we demonstrated that pneumonia caused by experimental infection with *C. gattii* was more severe in C57BL/6 TLR9^-/-^ mice than C57BL/6 WT mice. Although WT mice were also susceptible to *C. gattii*, TLR9-deficient mice had severe bronchiolitis and vasculitis, greater cellular infiltrate with eosinophils prevalence, diffuse interstitial pulmonary fibrosis and generalized alveolar destruction, indicating an important role of TLR9 for the protective immune response against *C. gattii*.

Some works have shown the importance of TLR9 activation in opportunistic fungi, as well as *C. neoformans, C. albicans* and *Aspergillus fumigatus* [36,37,38,39,54,55], but the importance of this receptor in the infection caused by *C. gattii* is still poorly understood.

Many works have already demonstrated a large cellular infiltrate in experimental cryptococcosis [15,24,25,26]. A high frequency of PMN cells close to vessels, arterioles and bronchioles for a prolongated and intense inflammatory response has been frequently associated with pneumonia caused by *Cryptococcus* spp. [24,25,29,30]. Pulmonary eosinophilia caused by cryptococcal infection has been associated with inefficiency in controlling or resolving the pulmonary infection, culminating in a Th2 allergic response [29,30,31]. Although *C. gattii*-infected WT mice had chronic cryptococcal pneumonia and pulmonary eosinophilia 21 days after inoculum, we found hypereosinophilia in TLR9^-/-^ pulmonary mixed infiltrate. TLR9-deficient mice had a high frequency of eosinophils in the lungs in comparison to WT mice 21 days post infection, while neutrophils were more frequent in the lungs of WT infected mice. High amounts of eosinophils in the lungs have already been described in murine experimental models using *C. neoformans* infection [24,25,29,30,31]. In addition, the IL-4, IL-5 and Th2 responses associated with eosinophilia have been described in *C. neoformans* pneumonia [24,25,26], but little is known about these populations during *C. gattii* infection. Eosinophilia in TLR9^-/-^ infected mice could be associated to the Th2 profile induced by *C. gattii*. Human PBMC produced large amounts of IL-4 after in vitro stimulation by heat-killed *C. gattii* [56]. C57BL/6 mice infected by the *C. gattii* hypervirulent strain had reduced Th1/Th17 cytokine expression and enhanced Th2 immune response [15]. CD1, BALB/c and C57BL/6 mice have shown different cytokine profiles when infected by *C. neoformans* or *C. gattii* [20,41,57]. Probably, this immune response polarization is the key to host susceptibility.

Recently characterized, cryptococcal titan cells are an important virulence factor and are associated with host susceptibility [42,43,58]. Many environmental factors linked to host immune response could be necessary for cryptococcal yeast enlargement. In the present study, we showed that cryptococcal titan cells were observed in both WT and TLR9^-/-^ mice 21 days after infection by *C. gattii*. Previously, we demonstrated that TLR9^-/-^ mice had a higher frequency of cryptococcal titan cells and produced lower amounts of IFN-γ and IL-17 than WT infected mice [41]. It was already shown that the appearance of *C. neoformans* titan cells is associated with high levels of Th2 cytokines and IgE [57], while the Th1/Th17 immune response is critical to control infection [15,16,17,18]. The abnormal growth of yeasts is considered an important fungal escape mechanism, since their phagocytosis by immune cells is hampered [44], in addition to increasing virulence [45]. Recently, Trevijano-Contador and colleagues (2022) showed that IL-17 played an important role in *C. neoformans* experimental infection [59]. Curiously, C57BL6 IL-17^-/-^ had less cryptococcal titan cells than WT infected mice 9 days after inoculum with the hypervirulent *C. neoformans* strain, H99. Furthermore, TNF-α intraperitoneal administration was able to attenuate the growth of cryptococcal yeasts in the lungs 48h after inoculum of the H99 strain. Apparently, the Th1/Th17 balance is fundamental for the establishment of infection by *C. neoformans*. Immunopathological aspects are different between *C. neoformans* and *C. gattii* infection in murine models. In a previous study of our group, we demonstrated that lower amounts of IFN-γ and IL-17 were associated with high titan cell frequency and severe lung impairment [41]. Furthermore, C57BL/6 mice had suppressed Th1 and Th17 immune responses and increased Th2 immune response as the *C. gattii* inoculum was increased during *C. neoformans* coinfection [15].

The importance of alveolar epithelial cells and Th2 response during a chronic inflammatory response is already well-described. The role of alveolar epithelial cells has been extensively explored in murine models of cryptococcal pneumonia. Heyen and colleagues (2016) demonstrated that type 2 alveolar epithelial cells were the major source of IL-33 during experimental infection using *C. neoformans* [23]. Type 2 alveolar epithelial cells support prolonged and disseminated pulmonary *C. neoformans* infection through IL-33 signaling [27,28]. Some research has demonstrated that type 2 alveolar epithelial cells constitutively express IL-33 and can be an amplifier of type 2 immune response [60,61]. Surfactant protein D (SPD) and IL-5 are also very important during *C. neoformans* infection. SPD exacerbates the cryptococcal pathogenicity and drives a non-protective host immune response associated with pulmonary eosinophilia [25]. However, little is known about IL-5, IL-33 and type 2 alveolar epithelial cells in the course of *C. gattii* experimental infection. In our model, we observed hyperplasia of type 2 alveolar epithelial cells (main source of SPD in the lung) in both WT and TLR9^-/-^ infected mice 21 days after *C. gattii* inoculum, which is associated with a mice model of pulmonary fibrosis [62] and idiopathic pulmonary fibrosis [53]. Apparently, the Th2 allergic response and type 2 alveolar epithelial cell hyperplasia are linked and constitute the complex immunopathology of cryptococcal infections.

Fibrosis in murine experimental infection with *Cryptococcus* spp. is little explored. However, chronic diseases with a pulmonary Th2 immune response, such as asthma, may help in identifying possible mechanisms involved in cryptococcal pneumonia. Pulmonary eosinophilia and high amounts of IL-4, IL-5, IL-13 and IgE in the lungs and serum are also characteristic of asthma [32]. The importance of IL-33 and alveolar epithelial cells in asthma is well-described [63,64,65]. Furthermore, some studies have shown that alveolar epithelial cells and IL-33 play an important role in wound healing and tissue repair [63,66]. Pulmonary fibrosis was observed in *C. neoformans*-infected IFN-γ^-/-^ mice. The Th1 immune response compromised in IFN-γ absence during *C. neoformans* infection led to Th2 immune response predominance, pulmonary eosinophilia, high levels of serum IgE and pulmonary fibrosis [29]. In the present study, our histological sections stained with picrosirius red evidenced a greater amount of thick fibrous elements in the lungs of infected TLR9^-/-^ mice compared to infected WT mice after 21 days after inoculum. Large areas with intense birefringence were observed in histological sections of TLR9^-/-^ infected mice. Little is known about lung fibrosis during cryptococcal infection, but our results showed that eosinophilia and fibrosis were associated with TLR9^-/-^ absence in the course of *C. gattii* infection.

Together, our results indicate that TLR9^-/-^ mice were more susceptible to *C. gattii* experimental infection in comparison to WT. A high number of eosinophils in pulmonary mixed cellular infiltrate, large interstitial pulmonary fibrosis and type 2 alveolar epithelial cell hyperplasia support the hypothesis that TLR9 plays an important role in controlling the infection at the primary site.

## 4. Materials and Methods

### 4.1. Cryptococcus Strain

*Cryptococcus gattii* R265 strain (Serotype B), hypervirulent, VGIIa molecular type, with alpha mating type, was kindly provided by Professor Leonardo Nimrichter (Instituto de Microbiologia Paulo Góes, Universidade Federal do Rio de Janeiro, Brazil). Yeasts were cultured in a liquid defined medium (Sabouraud’s medium) at 30 °C with continuous shaking (100 rpm) for 2 days in 10 mL. A total of 500 μL was collected and cultivated in 20 mL of Sabouraud’s liquid medium for 2 days. After, they were cultured for more 5 days in liquid minimal medium [67].

### 4.2. Inoculum Preparation

Fungal culture in liquid medium (1 mL) was collected and centrifuged at 10,000 rpm for 3 min. The pellet was resuspended in 30 mL of sterile PBS and centrifuged twice under the same conditions. The pellet was resuspended once more in 1 mL of sterile PBS. After, we counted the yeasts on a Neubauer chamber. An infection inoculum of 10^4^ yeast in 30 µL of PBS was used [68].

### 4.3. Mice and Infection Model

Isogenic mice of the C57BL/6 (WT) and C57BL/6 TLR9^-/-^ (TLR9^-/-^) [69] strain, male, aged 8–10 weeks, weighing between 25 g and 30 g, were used in this study. The C57BL/6 TLR9^-/-^ mice were kindly donated by the Laboratório de Imunofarmacologia, Centro de Ciências da Saúde, Instituto de Biofísica Carlos Chagas Filho, Universidade Federal do Rio de Janeiro, RJ, Brazil, and the C57BL/6 WT line was kindly donated by the Instituto de Veterinária, Departamento de Microbiologia e Imunologia Veterinária, Universidade Federal Rural do Rio de Janeiro, RJ, Brazil. The animals were maintained in sterile (grouped) cages, under standardized conditions of temperature (22–23) °C and light (cycles of 12 h of light and 12 h of dark), with commercial feed and drinking water provided ad libitum. The use of the animals in this study was approved by the Ethics Committee on the Use of Animals (CEUA) at UFRJ (Nº:092/21). The mice were sacrificed according to the criteria approved by CEUA at the time of the study. All animal work was performed in accordance with Animal Research: Reporting of In Vivo Experiments (ARRIVE) guidelines and regulations.

### 4.4. Anesthesia and Analgesia

Prior to intratracheal infection, anesthesia and analgesia of the animals were performed intraperitoneally with Xylazine (10 mg/kg) and Ketamine (20 mg/kg) in each animal.

### 4.5. Intratracheal Infection

The animals were subjected to intratracheal infection with 10^4^ encapsulated yeast cells of *C. gattii* (R265 strain) in a total volume of 30 µL/animal, with sterile PBS as the vehicle. Uninfected (Sham) groups were given 30 µL of sterile PBS only.

### 4.6. Histological Sections

After euthanasia, the lungs were excised. Both lungs were placed in identified cassettes and immersed in formaldehyde (Sigma-Aldrich, St. Louis, MO, USA) at 10% for 24 h. After, they were placed in 70% ethanol and processed. Initially, for the diaphanization stage, tissues were transferred to two baths of 100% alcohol. They were then immersed in two xylol baths followed by two baths in liquefied paraffin. Thick cuts of 5–6 µm of the tissues in paraffin molds were obtained with the aid of a microtome. The sections were stained by picrosirius red [70], Congo red [71], hematoxylin–eosin or Alcian Blue, pH 2.5 [72]. The slides were mounted on Enthelan (Sigma-Aldrich). The slides were analyzed under light microscopy (Leica DM750, Wetzlar, Germany), and the images were captured using a Leica DFC425 digital camera. For histological sections, four infected WT and three infected TLR9^-/-^ mice were used; three mice for each Sham group (WT and TLR9^-/-^) were also used.

### 4.7. Eosinophils and Neutrophils Frequency

Eosinophils and neutrophils were counted in random fields of histological sections stained with Congo red. Each lung (right and left) was divided into four quadrants, and a random field was used to count 100 polymorphonucleated cells (eosinophils and neutrophils). After counting the cells in random fields of the four quadrants, the arithmetic mean was calculated, and the value referring to the cell frequency of each lung was obtained. The mean represents the arbitrary value referring to the frequency of eosinophils and neutrophils (in 100 cells) of each mouse.

### 4.8. Statistical Analysis

Statistical analyses were performed using the GraphPad Prism 5.0 program, with the Student’s *t*-test and Mann–Whitney U test (as appropriate). Values of *p* ≤ 0.05 indicate statistical significance, with significant differences designated as *** *p* ≤ 0.001, ** *p* ≤ 0.01 and * *p* ≤ 0.05.

## Data Availability

The data is contained within this article.
